# Baicalein inhibits the pharmacokinetics of simvastatin in rats *via* regulating the activity of CYP3A4

**DOI:** 10.1080/13880209.2021.1942927

**Published:** 2021-07-02

**Authors:** Meng Meng, Xin Li, Xiuwen Zhang, Bin Sun

**Affiliations:** a Department of Cardiovascular Medicine, Yidu Central Hospital of Weifang, Weifang, Shandong, China; b Department of Nursing, Yidu Central Hospital of Weifang, Weifang, Shandong, China; c Department of Critical Care Medicine, Yidu Central Hospital of Weifang, Weifang, Shandong, China; d Department of Emergency, Yidu Central Hospital of Weifang, Weifang, Shandong, China

**Keywords:** Drug-drug interaction, *Scutellariae radix*, metabolism, biotransformation

## Abstract

**Context:**

Baicalein and simvastatin possess similar pharmacological activities and indications. The risk of their co-administration was unclear.

**Objective:**

The interaction between baicalein and simvastatin was investigated to provide reference and guidance for the clinical application of the combination of these two drugs.

**Materials and methods:**

The pharmacokinetics of simvastatin was investigated in Sprague–Dawley rats (*n* = 6). The rats were pre-treated with 20 mg/kg baicalein for 10 days and then administrated with 40 mg/kg simvastatin. The single administration of simvastatin was set as the control group. The rat liver microsomes were employed to assess the metabolic stability and the effect of baicalein on the activity of CYP3A4.

**Results:**

Baicalein significantly increased the AUC_(0–_*_t_*_)_ (2018.58 ± 483.11 *vs.* 653.05 ± 160.10 μg/L × h) and *C*_max_ (173.69 ± 35.49 *vs.* 85.63 ± 13.28 μg/L) of simvastatin. The t_1/2_ of simvastatin was prolonged by baicalein *in vivo* and *in vitro*. The metabolic stability of simvastatin was also improved by the co-administration of baicalein. Baicalein showed an inhibitory effect on the activity of CYP3A4 with the IC_50_ value of 12.03 μM, which is responsible for the metabolism of simvastatin.

**Discussion and conclusion:**

The co-administration of baicalein and simvastatin may induce drug-drug interaction through inhibiting CYP3A4. The dose of baicalein and simvastatin should be adjusted when they are co-administrated.

## Introduction

Cardiovascular and cerebrovascular diseases are responsible for a large part of mortality (Izzo et al. 2018). Simvastatin is one of the major drugs in the clinical treatment of cardiovascular and cerebrovascular diseases with excellent hypolipidemic effects (Mendes et al. 2014). As the development of cardiovascular and cerebrovascular diseases is a complex process involving various complications, the therapy of cardiovascular and cerebrovascular diseases always combines Chinese and Western medicines to improve treatment efficiency and reduce side effects (Zhen et al. 2014; Lendahl et al. 2019). The pharmacokinetics of simvastatin that depends on its metabolism and absorption is the main factor that affected the curative effectiveness (Jiang et al. 2017). In a previous study, the combination of simvastatin and berviscapine increased the plasma concentration of simvastatin by inhibiting the mRNA expression of the hepatic CYP3A4 (Ju et al. 2017).

Baicalein is a wildly used herbal medicine derived from *Scutellariae baicalensis* Georgi (Lamiaceae), which possesses various pharmacological activities, such as antioxidant, anti-inflammatory, and antimicrobial, and it also has been demonstrated to improve blood circulation, increase cerebral blood flow, and anti-platelet aggregation (Lin and Shieh 1996; Liang et al. 2017). Baicalein is the major component responsible for the pharmacological effects of *S*cutellariae *radix* (Li, Lin, et al. 2011). Baicalein is always co-administrated with cardiovascular and cerebrovascular drugs. It was reported that baicalein combined with nimodipine enhances the oral bioavailability of nimodipine via the inhibition of P-glycoprotein (P-gp) and CYP3A4 (Cho et al. 2011). Therefore, the combination of baicalein with simvastatin may be applied in the treatment of cardiovascular and cerebrovascular diseases.

Hence, ascertaining the interaction between baicalein and simvastatin is of great significance, which can provide reference and guidance for the clinical co-administration of these two drugs. This study investigates the effect of baicalein on the pharmacokinetics of simvastatin in rats and evaluates the risk of the co-administration of baicalein and simvastatin.

## Materials and methods

### Chemicals and animals

Simvastatin was obtained from Shandong Lukang Pharmaceutical Co. (Shandong province, China) and baicalein was purchased from Sigma Chemical Co. (St. Louis, MO, USA). The methanol, acetonitrile, and acetic acid were obtained from Merck Co. (Darmstadt, Germany) with the purity of HPLC grade.

Male Sprague–Dawley rats (230–250 g) were obtained from Sino-British Sippr/BK Lab Animal Ltd (Shanghai, China). The rats were housed by a 12 h light-dark cycle at 23 ± 2 °C with a relative humidity of 50–60%. Rats were fasted for 12 h and had free access to water before experiments. This study was approved by the Animal Care and Use Committee of Yidu Central Hospital of Weifang.

### Pharmacokinetic study in rats

The rats were divided into two groups randomly, including a control group and a test group, with six rats of each group. The rats in the control group were orally administrated with 40 mg/kg simvastatin and the test group was pre-treated with 20 mg/kg baicalein for 10 d, and then administrated with 40 mg/kg simvastatin. The dose of simvastatin and baicalein was used to refer to previous studies (Noh et al. 2015; Jiang et al. 2017). Simvastatin and baicalein were administrated separately to avoid the chemical interaction between these two drugs. After 0, 0.083, 0.25, 0.5, 1, 2, 3, 4, 6, 8, 12, and 24 h of simvastatin administration, the plasma samples were collected and centrifuged for the analysis of simvastatin with the help of HPLC.

### LC-MS/MS condition

Lovastatin was used as the internal standard (IS) for the analysis of simvastatin. The analysis was conducted with Agilent 1290 series liquid chromatography system and an Agilent 6460 triple-quadrupole mass spectrometer (Palo Alto, CA, USA). Water and acetonitrile were used as the mobile phase with a flow rate of 0.3 mL/min and an analysis time of 4 min. The *m/z* ratios of precursor-to-product ion reactions for simvastatin and IS were 419.32/199.15 and 405.3/199.15, respectively.

### Metabolic stability study in rat liver microsomes

An NADPH-generating system was preincubated for 5 min and then mixed with simvastatin. For the test group, baicalein was added before simvastatin and preincubating for 30 min. The metabolic stability was evaluated by 0, 1, 3, 5, 15, 30, and 60 min of incubation with the following equations:

$$t_{1/2}=0.693/k;$$

V(μL/mg)=volume of incubation (μL)/protein in the incubation (mg);

$$\text{Intrinsic}\;\text{clearance}\;\left(\text{Clint}\right)\;\left(\mu L/\text{min}/\text{mg}\;\text{protein}\right)=V\times 0.693/t_{1/2}$$

### CYP3A4 inhibition assay

To evaluate the inhibition of CYP3A4 enzyme activity, the interaction was performed with rat liver microsomes, typical CYP3A4 substrates (testosterone), and various concentrations of baicalein, according to the previous study (Ding et al. 2020; Li et al. 2020). The activity of CYP3A4 was evaluated by the concentration of substrates with the employment of HPLC.

### Statistical analysis

All detection in the study was performed at least in triplicate and analyzed by Graphpad or SPSS. The data comparison was conducted by one-way ANOVA. The difference was considered to be statistically significant when *p* < 0.05. The pharmacokinetic parameters were calculated with DAS 3.0 pharmacokinetic software.

## Results

### Effect of baicalein on the pharmacokinetic profile of simvastatin

**Figure 1.**
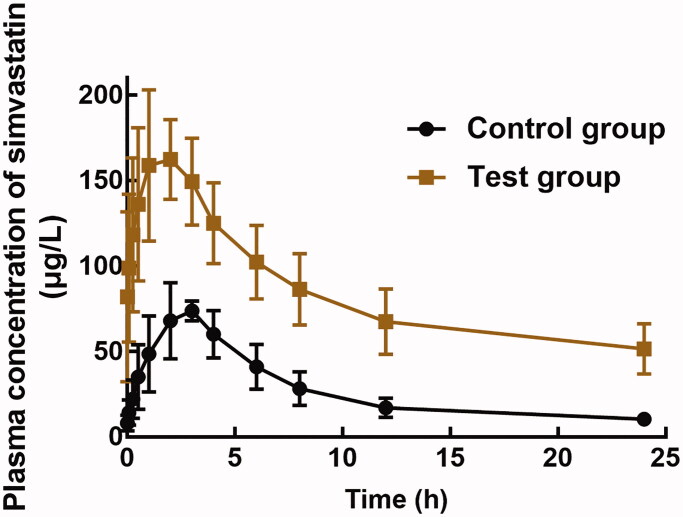
The plasma concentration-time curve of 40 mg/kg simvastatin in the presence (test group) or absence (control group) of 20 mg/kg baicalein.

The plasma concentration-time curve of simvastatin in the presence or absence of baicalein is shown in Figure 1. The pharmacokinetic profile of simvastatin was significantly changed by the administration of baicalein. As summarized in Table 1, the AUC_(0–_*_t_*_)_ of simvastatin increased from 653.05 ± 160.10 to 2018.58 ± 483.11 μg/L × h in the presence of 20 mg/kg baicalein. Consistently, the *C*_max_ increased to 173.69 ± 35.49 μg/L with the co-administration of baicalein. Additionally, baicalein also prolonged the *t*_1/2_ (4.89 ± 1.04 h to 10.18 ± 2.43 h) of simvastatin and advanced the arrival of *C*_max_ (*T*_max_ from 2.83 ± 0.75 to 1.83 ± 0.62 h). These results indicate increased system exposure of simvastatin when co-administrated with baicalein.

**Table 1.** The pharmacokinetic parameters of 40 mg/kg simvastatin in rats with (test group) or without (control group) the pre-treatment of 20 mg/kg baicalein.

	Control group	Test group
AUC_(0–_*_t_*_)_ (μg/L×h)	653.05 ± 160.10	2018.58 ± 483.11
*t*_1/2_ (h)	4.89 ± 1.04	10.18 ± 2.43
*T*_max_ (h)	2.83 ± 0.75	1.83 ± 0.62
*C*_max_ (μg/L)	85.63 ± 13.28	173.69 ± 35.49
Clz/F (L/h/kg)	63.80 ± 23.39	17.39 ± 5.36

### Effect of baicalein on the metabolic stability of simvastatin

In rat liver microsomes, the half-life (*t*_1/2_) of simvastatin was 33.51 min with the intrinsic clearance rate of 41.36 μL/min/mg protein. In the presence of baicalein, the *t*_1/2_ of simvastatin increased to 47.56 min and the intrinsic clearance rate decreased to 29.14 μL/min/mg protein, indicating the improved metabolic stability of simvastatin by baicalein.

### Effect of baicalein on the activity of CYP3A4

**Figure 2.**
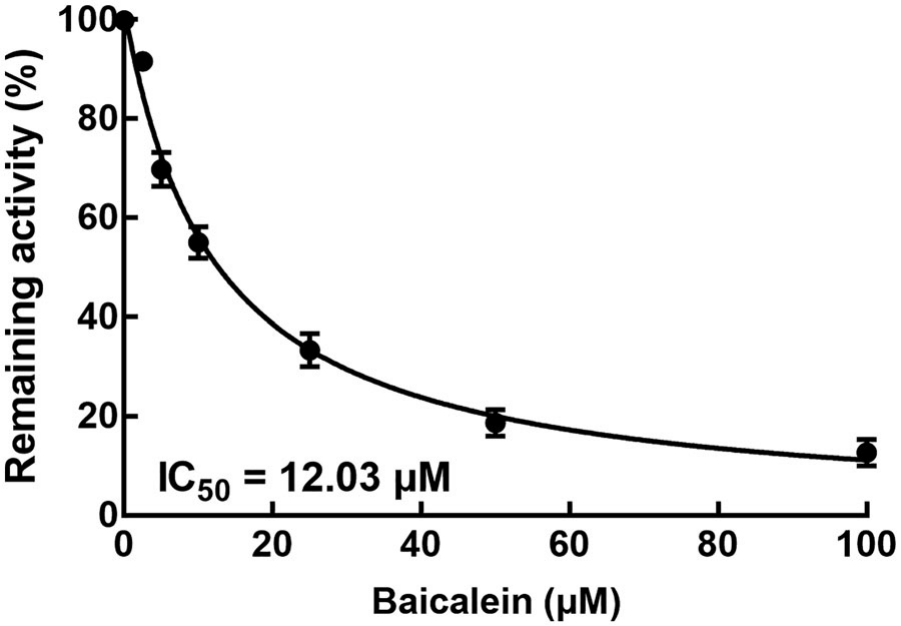
Effect of baicalein on the activity of CYP3A4. The activity of CYP3A4 was evaluated by detecting the concentration of specific metabolites in the presence of 0, 2.5, 5, 10, 25, 50, and 100 μM baicalein.

The potential mechanism underlying the effect of baicalein on the pharmacokinetics of simvastatin was further evaluated in rat liver microsomes. As shown in Figure 2, the activity of CYP3A4 significantly decreased with the increasing concentration of baicalein, and the IC_50_ value of CYP3A4 by baicalein was obtained as 12.03 μM, suggesting the significant inhibitory effect of baicalein on the activity of CYP3A4.

## Discussion

To improve the efficiency of disease treatment, it is common to combine different drugs in the clinic (Zhang et al. 2010; Wang et al. 2017). Co-administration of different drugs might lead to therapy failure and even toxicity resulting from interactions between co-administrated drugs. For example, the combination of peimine and paeoniflorin for the treatment of cough could cause the increased plasma concentration of paeoniflorin in rats implying the drug-drug interaction between peimine and paeoniflorin (Chen et al. 2021). The co-intravenous administration of Shuanghuanglian and azithromycin could increase the drug exposure of each other and might improve the therapy efficiency of these two injections (Tian et al. 2019). Baicalein and simvastatin are two important herb and drug in the prescription of cardiovascular and cerebrovascular diseases in the clinic due to their similar indications, including hyperlipidemia, coronary heart disease, and cerebrovascular diseases (Collins et al. 2004; Chen et al. 2018; Shi et al. 2018; Yang et al. 2019). Whether the co-administration of these two drugs could induce drug-drug interaction is still unclear, which is important for the clinical application of these two drugs.

Baicalein was found to change the pharmacokinetic profile of simvastatin and increase the AUC of simvastatin and prolonged the *t*_1/2_ in rats, indicating the increasing system exposure of simvastatin. CYP3A4 is one of the major isoforms of cytochrome P450 enzymes, which is responsible for first-pass metabolism and bioavailability of numerous drugs and herbs (Manikandan and Nagini 2018). The effect of different drugs on the activity of CYP3A4 could induce adverse drug–drug interactions that are closely associated with the drug biotransformation and therapeutic efficiency (Zhou 2008). In a previous study, baicalein was reported to inhibit the clearance of tamoxifen by inhibiting CYP3A4 and P-gp and therefore increase the drug bioavailability (Li, Kim, et al. 2011). Here, the inhibitory effect of baicalein on the activity of CYP3A4 was also validated in rat liver microsomes. It was found that baicalein dramatically inhibited the activity of CYP3A4 in a dose-dependent manner with the specific IC_50_ value. It has been demonstrated that simvastatin is highly selective to the liver, where its concentration is significantly higher than in other tissues (Pedersen and Tobert 2004). CYP3A4 is widely distributed in the liver and contributes to the metabolism of simvastatin (Kitzmiller et al. 2014). Hence, it was speculated that the effect of baicalein on the pharmacokinetics of simvastatin was a result of the inhibition of CYP3A4.

## Conclusions

Taken together, baicalein increased the system exposure and the metabolic stability of simvastatin in rats. The potential mechanism might be the inhibition of CYP3A4. While the metabolism of simvastatin involves various factors and processes, the inhibition of CYP3A4 might be one possible reason for those factors. Therefore, the drug-drug interaction between simvastatin and baicalein needs to be validated *in vivo* and the mechanism can be explored more widely.
